# Type 2 diabetes and its characteristics are associated with poor oral health: findings from 60,590 senior women from the E3N study

**DOI:** 10.1186/s12903-021-01679-w

**Published:** 2021-06-23

**Authors:** Nasser Laouali, Douae El Fatouhi, Gloria Aguayo, Beverley Balkau, Marie-Christine Boutron-Ruault, Fabrice Bonnet, Guy Fagherazzi

**Affiliations:** 1 Center for Research in Epidemiology and Population Health (CESP), Inserm (Institut National de la Santé Et de la Recherche Médicale) U1018, Generations and Health, Gustave Roussy Institute, 114 rue Edouard Vaillant, 94805 Villejuif Cedex, France; 2 Faculté de Médecine, UPS-UVSQ-Paris-Saclay University, 94270 Le Kremlin-Bicêtre Cedex, France; 3 Deep Digital Phenotyping Research Unit, Department of Population Health, Luxembourg Institute of Health, 1A-B, rue Thomas Edison, 1445 Strassen, Luxembourg; 4 Center for Research in Epidemiology and Population Health (CESP), Inserm (Institut National de la Santé et de la Recherche Médicale) U1018, Clinical Epidemiology, 16 Avenue Paul Vaillant Couturier, 94807 Villejuif, France; 5 Department of Endocrinology, Diabetology and Nutrition, CHU Rennes, Université de Rennes 1, Rennes, France

**Keywords:** Periodontitis, Gingivitis, Treatments, Aged, Female, Type 2 diabetes mellitus, Epidemiology

## Abstract

**Background:**

Type 2 diabetes (T2D) has been identified as a risk factor for poor oral health, however, a limited number of oral health and T2D characteristics have been studied so far. We sought to assess T2D status, age at diagnosis, duration since diagnosis and treatment in relation to a variety of oral diseases.

**Methods:**

Cross-sectional data were analyzed from the E3N (Etude Epidémiologique auprès de femmes de l'Education Nationale) cohort study which enrolled 60,590 women. Participants self-reported oral health status, and T2D cases were identified using diabetes-specific questionnaires and drug reimbursement insurance databases. Multivariable-adjusted ORs and 95% CIs were estimated using logistic regression models.

**Results:**

The mean age (SD) of the women was 70 years (7.2), and 4.7% (n = 2857) had T2D. Compared to women without T2D, women with T2D were more likely to report a poor perceived oral health (OR 1.37 [95% CI 1.18, 1.60]), wearing dental prostheses (1.26 [1.14, 1.39]) and having problems of biting and chewing food (1.19 [1.07, 1.33]). In addition, for women with T2D the age at diagnosis (inversely) and the duration (positively) were associated with the likelihood to report poor oral health.

**Conclusions:**

For women with T2D, duration and age at diagnosis are associated with wearing prostheses, problems of biting and chewing, periodontitis and gingivitis.

**Supplementary Information:**

The online version contains supplementary material available at 10.1186/s12903-021-01679-w.

## Background

Oral diseases are among the most common forms of chronic disease, with a strong effect on self-esteem, quality of life and overall health and well-being [1]. Although oral health is often uncared for in general health [1, 2], the World Health Organization (WHO) estimates that oral diseases affected fifty per cent of the global population with untreated dental caries and severe periodontal disease being the most prevalent diseases [1–3]. While all the underlying causes of oral health have not yet been identified, several risk factors have been suggested and may help our understanding of the fundamentals of oral disease pathophysiology. Among them, oral hygiene and smoking are by far the most important determinant, but diabetes has also been shown to play a critical role [4, 5].

Indeed, type 2 diabetes is a chronic disease that has concomitant oral manifestations that affect dental care. Elevated levels of pro-inflammatory mediators in poorly controlled diabetes play a role in the elevated risk of oral diseases such as periodontal destruction [6, 7]. For example, periodontal diseases (such as periodontitis and gingivitis) have been listed as the sixth most prevalent complication of diabetes [8]. It has also been reported that oral health may have an impact on type 2 diabetes risk and its metabolic control [1–3, 9], suggesting a complex bidirectional link between type 2 diabetes and oral diseases. However, there is more evidence that type 2 diabetes precedes periodontal diseases than the reverse [10, 11]. Previous data reported that diabetes duration and severity are positively associated with high decayed, missed and filled teeth values [12], periodontal diseases [13, 14], tooth loss [15] and lower scores of oral health related quality of life [16]. In contrast, a recent meta-analysis reports a non-significant association between poorly controlled type 2 diabetes and the risk of periodontitis as well as a high level of heterogeneity between studies [17]. The conflicting results highlight the need for further studies and there is no data in French population. In addition, while oral health is a general concept, a limited number of its characteristics have been used as outcomes in previous studies and no previous work has studied the various characteristics of oral health concomitantly. Furthermore, despite the gender differences in diabetes incidence [18] and the burden of age-related oral health [19], there are very few gender and age-specific studies.

Therefore, based on the detailed information from the large female E3N (Etude Epidémiologique auprès de femmes de l'Education Nationale) cohort study, we aim to evaluate the associations between type 2 diabetes and its characteristics (age at diagnosis, duration since diagnosis and treatment use) and oral health components in older women. Our hypothesis is that older women with type 2 diabetes are more likely to report poor oral health compared to older people without diabetes. In addition, diabetes duration and age at diagnosis are correlated with the likelihood of reporting poor oral health.

## Methods

### Cohort and study population for analysis

Women included in the present study are from the E3N cohort, a large ongoing French prospective cohort study, set up in 1990. The study was performed in accordance with the Declaration of Helsinki and received ethical approval from the French National Commission for Computerized Data and Individuals (CNIL); all participants gave written informed consent. The detailed protocol has been described elsewhere [20] and registered at clinicaltrials.gov as NCT03285230. Briefly, 98,995 women born between 1925 and 1950 were selected from the French national health insurance plan for teachers and coworkers, the Mutuelle Générale de l'Education Nationale. Data were collected at inclusion and every 2 years by self-administered questionnaires. Information recorded included health conditions, lifestyle, diet, treatments, etc. Furthermore, all outpatient reimbursements for health expenditure since January 1, 2004; of each participant were determined through a health insurance plan. These data included brand names, doses, and dates of drug reimbursements. The average response rate to a follow-up questionnaire is 83%, with a total loss to follow-up between 1990 and 2011 (date of wave 10 questionnaire which had items on oral health) was below 3%.

This study is a cross-sectional analysis of the E3N 10th wave questionnaire where 70,592 women completed information on oral health. From this sample, we further excluded all participants with missing data on one or more oral health items (n = 9152) and all incident type 2 diabetes cases that occurred after December 07, 2011, date of the 10th wave questionnaire return (n = 852). The final study population included 60,590 women (see Additional file 1: Fig. 1).

**Fig. 1:**
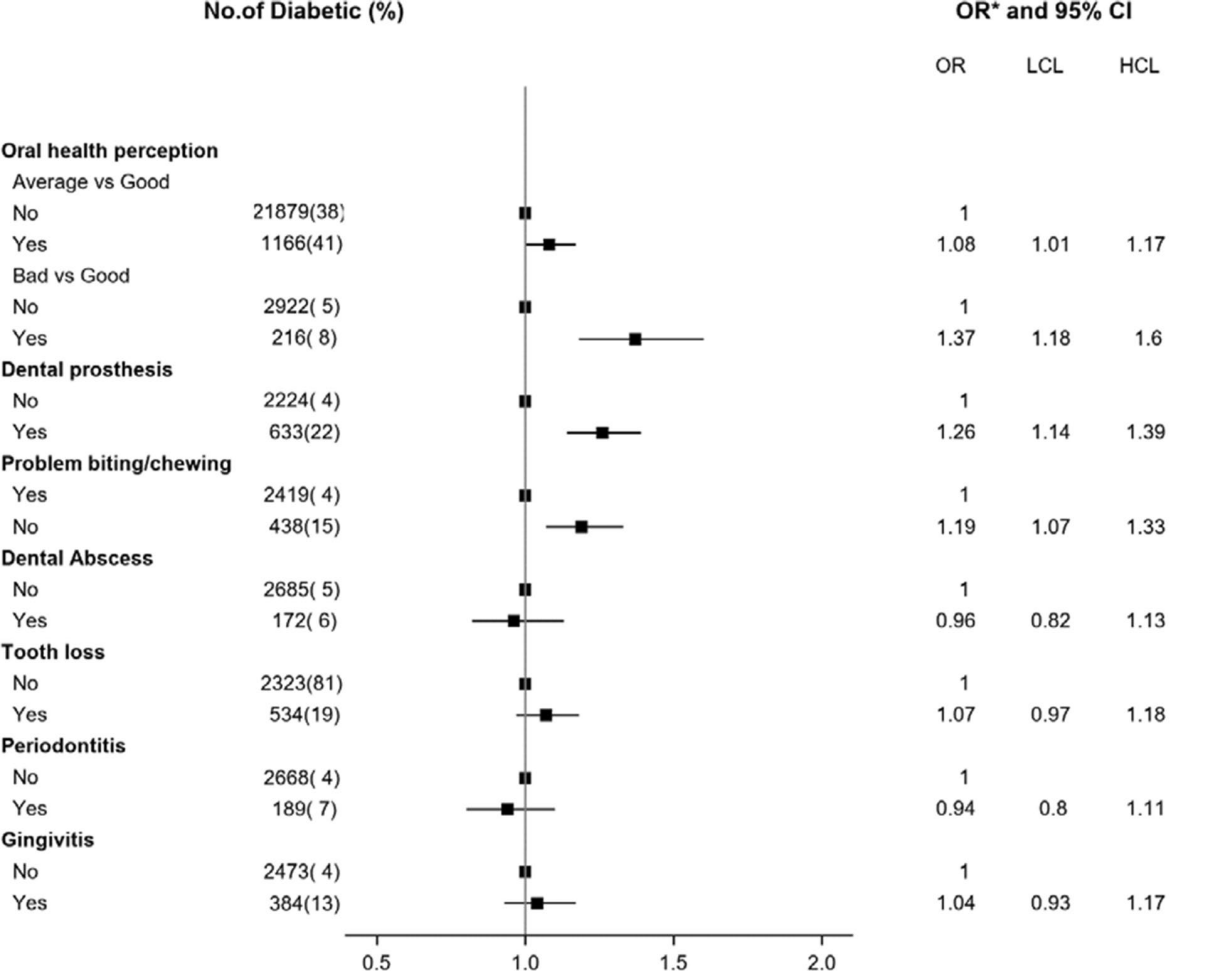
Odds ratios of perceived oral health (yes/no) comparing women with type 2 diabetes to those without type 2 diabetes. LCI, low confidence interval; UCI, upper confidence interval; * Adjusted for age, educational level, smoking status, physical activity, family history of diabetes, hypercholesterolemia, hypertension, body mass index, dietary inflammatory index, daily brushing frequency and annual frequency of visit to the dentist

### Ascertainment of type 2 diabetes and its characteristics

The detailed procedure has been described in detail elsewhere [21]. Subsequently, a diabetes-specific questionnaire was sent to all potential cases identified through follow-up questionnaires. In order to be considered as validated, a potential case must have reported at least one of the following (1) fasting plasma glucose ≥ 7.0 mmol/l or random glucose ≥ 11.1 mmol/l at diagnosis; (2) use of a glucose-lowering medication; (3) values of fasting glucose or HbA1c concentrations ≥ 7.0 mmol/l or ≥ 7%, in the diabetes-specific questionnaire. After 2004, cases identification was based on the drug reimbursement insurance database. All women with at least two reimbursements for any glucose-lowering medications during 1 year were considered to have validated diabetes, with the date of diagnosis defined as the date of first reimbursement. For each validated case of type 2 diabetes, age at diagnosis, duration and treatment use were identified.

Age at diagnosis was calculated as the difference between the date of diagnosis and the date of birth. Diabetes duration was defined as the difference between December 12, 2011, date of wave 10 questionnaire return and the date of diagnosis. Treatment use considered was the last glucose lowering drug(s) [oral antidiabetic agents (OAD), insulin] used in the 6 months prior to the 10th wave questionnaire return. Anti-diabetic agents used were classified as OAD or insulin or the combination of OAD and insulin. Cases with no pharmacological treatment were also identified.

### Assessment of perceived oral health

Oral health status and behaviors were self-reported only in the 10th wave questionnaire (December 12, 2011) in nine items adapted from the existing validated WHO oral health questionnaire for adults translated in French [22]. The adapted questionnaire was not validated. The following outcomes were considered: overall perception of oral health: good oral health, average oral health, poor oral health, information on teeth: yes, no (tooth loss, dental abscesses, problems of biting and chewing, dental prostheses) as well as other conditions such as periodontitis and gingivitis diseases: yes, no. Daily brushing frequency and annual frequency of visits to the dentist were also considered as important characteristics of oral health and were used as adjusting factors in the modeling (see below).

### Covariables

In general, covariables used were measured at the 10th wave but if not available, we used the measurement at the closest wave for all women. BMI (wave 10 at 2011) was calculated by dividing weight in kilograms by height in meters squared and was considered as a continuous variable in all models. Age at wave 10, dietary inflammatory index [21] (wave 3 at 1993) and the level of recreational physical activity (wave 8 at 2005) (MET-h/week) were considered as continuous variables. Family history of diabetes (wave 8), personal history of hypertension (wave 9 at 2008) and hypercholesterolemia (wave 7 at 2002) were in two categories (yes and no). We considered three categories for smoking status (wave 10) (never, former, and current), educational level (wave 1 at 1990) (undergraduate or less, graduate, and postgraduate or more), and frequency of visits to the dentist (wave 10) (once a year or more, sometimes, and never). Daily brushing of teeth (wave 10) was considered in four categories (less than once, one, two, and three or more). Missing values were < 5% for all variables and therefore were imputed by the median and mode for quantitative and qualitative variables, respectively. We use simple imputation as we have empirically shown that results were most often unchanged when using more complex imputation techniques such as multiple imputation when analyzing E3N data [21]. There are few missing data for covariables, overall that the imputation technique does not have any impact on the results.

### Statistical analysis

The distributions of the study population characteristics overall and by type 2 diabetes status were expressed as means and standard deviation (SD) for continuous variables and as number (percentage) for categorical variables. For type 2 diabetes cases, the distributions of those characteristics were also reported by quartile of diabetes duration, age at diabetes diagnosis, and type of treatment use. Crude and multivariable unconditional logistic regressions were used to estimate associations between exposure variables (type 2 diabetes status, age at diagnosis, duration since diagnosis and treatment use) and outcome variables (self-reported oral health characteristics). For oral health perception considered in three levels (good, average and poor), we used multinomial logistic regression models. Odds ratios (OR) and their 95% confidence intervals (95% CI) were calculated in two different models: model 1 was unadjusted; model 2 was adjusted for age, educational level, smoking status, physical activity, family history of diabetes, hypercholesterolemia, hypertension, body mass index, dietary inflammatory index, daily brushing frequency and annual frequency of visits to the dentist.

In addition to our main analysis described previously, we tested for interactions between type 2 diabetes and BMI, smoking status and family history of diabetes as several studies draw a hypothetical picture of synergy between type 2 diabetes status and those variables in terms of damage to some oral diseases such as periodontitis [23–25]. As sensitivity analysis, we further adjusted model 2 for mentally tiring work (little or not, moderate, and high) assessed at wave 2 questionnaire (1992) and included as a categorical variable. Mentally tiring work, an indicator of work demands is a risk factor for several metabolic disorders and is associated with type 2 diabetes risk in our previous study in the E3N cohort [26].

All analyses were performed using Statistical Analysis Systems (SAS) software, version 9.3 (SAS Institute, Inc, Cary, NC, USA).

## Results

Overall, 4.7% (n = 2857) of the women had type 2 diabetes at wave 10. Table 1 presents characteristics of women, overall and according to type 2 diabetes status. The overall mean (SD) age of the women was 70.2 (6.2) years. Compared with women without type 2 diabetes, women with type 2 diabetes tended to be older and more frequently had hypertension, hypercholesterolemia and a family history of diabetes (Table 1). In addition, they were more likely to declare less than one daily brushing of teeth and not visiting a dentist annually. Characteristics of women according to quartile groups of duration and age at diagnosis of type 2 diabetes and type of treatment use are presented in Additional file 1: Tables s1–s3.

**Table 1:** Characteristics of the E3N study population, overall and according to diabetes status (N = 60,590 women), the E3N study

	All (N = 60,590)	Without type 2 diabetes (N = 57,733)	With type 2 diabetes (N = 2857)	*P*-value
Age (years)	70.17 (6.22)	70.07 (6.20)	72.27 (6.35)	< 0.0001
Educational level (%)				< 0.0001
Undergraduate or less	6791 (11)	6315 (10.94)	476 (16.66)	
Graduate	32,039 (53)	30,497 (52.82)	1542 (53.97)	
Postgraduate or more	21,760 (36)	20,921 (36.24)	839 (29.37)	
Physical activity (MET h/week)	59.56(47.70)	59.88 (47.86)	53.12 (43.99)	< 0.0001
Smoking status (%)				< 0.0001
Never	2855 (4.71)	2748 (4.76)	107 (3.75)	
Former	16,570 (27.35)	15,788 (27.35)	782 (27.37)	
Current	41,165 (67.94)	39,197 (67.89)	1968 (68.88)	
Dietary inflammatory index	0.12 (3.27)	0.14 (3.26)	-0.41 (3.30)	< 0.0001
BMI (kg/m^2^)	24.03 (3.95)	23.86 (3.78)	27.53 (5.43)	< 0.0001
Hypercholesterolemia (%)	6,830 (11)	6372 (11.04)	458 (16.03)	< 0.0001
Hypertension (%)	9928 (16)	9056 (15.69)	872 (30.52)	< 0.0001
Family history of diabetes (%)	7455 (12)	6672 (11.56)	783 (27.41)	< 0.0001
Daily brushing (%)				< 0.0001
Less than once	724 (1.20)	631 (1.09)	93 (3.26)	
One	12,298 (20)	11,505 (19.93)	793 (27.76)	
Two	29,533 (49)	28,190 (48.83)	1343 (47.01)	
Three or more	18,035 (30)	17,407 (30.15)	628 (21.97)	
Visits to the dentist (%)				0.0420
Once a year or more	43,300 (71.5)	41,358 (71.64)	1942 (67.97)	
Sometimes	16,050 (26.5)	15,234 (26.38)	816 (28.56)	
Never	1240 (2.0)	1141 (1.98)	99 (3.47)	

N (%) and *p*-value of the Chi-square test for categorical variables

Mean (SD) and *p*-value of the Student test for continuous variables

Percent of missing data: Smoking status (3.7), educational level (4.0), daily brushing (0.2), visits to the dentist (0.4), BMI (4.5) and physical activity (2.6)

### Type 2 diabetes status and perceived oral health

Figure 1 shows the OR of perceived oral health associated with type 2 diabetes. Women with type 2 diabetes were more likely to report a poor perceived oral health than women without type 2 diabetes [adjusted OR = 1.37 (95% CI 1.18 to 1.60)]. When the components of oral health outcome were further examined, women who had type 2 diabetes were more likely to self-report wearing dental prostheses [adjusted OR = 1.26 (95% CI 1.14 to 1.39)] and having problems of biting and chewing food [adjusted OR = 1.19 (95% CI 1.07 to 1.33)] compared to women without type 2 diabetes. In the fully adjusted model, dental abscesses, tooth loss, periodontitis and gingivitis were not associated with type 2 diabetes status (Fig. 1).

### Type 2 diabetes duration and age at diagnosis and perceived oral health

**Fig. 2:**
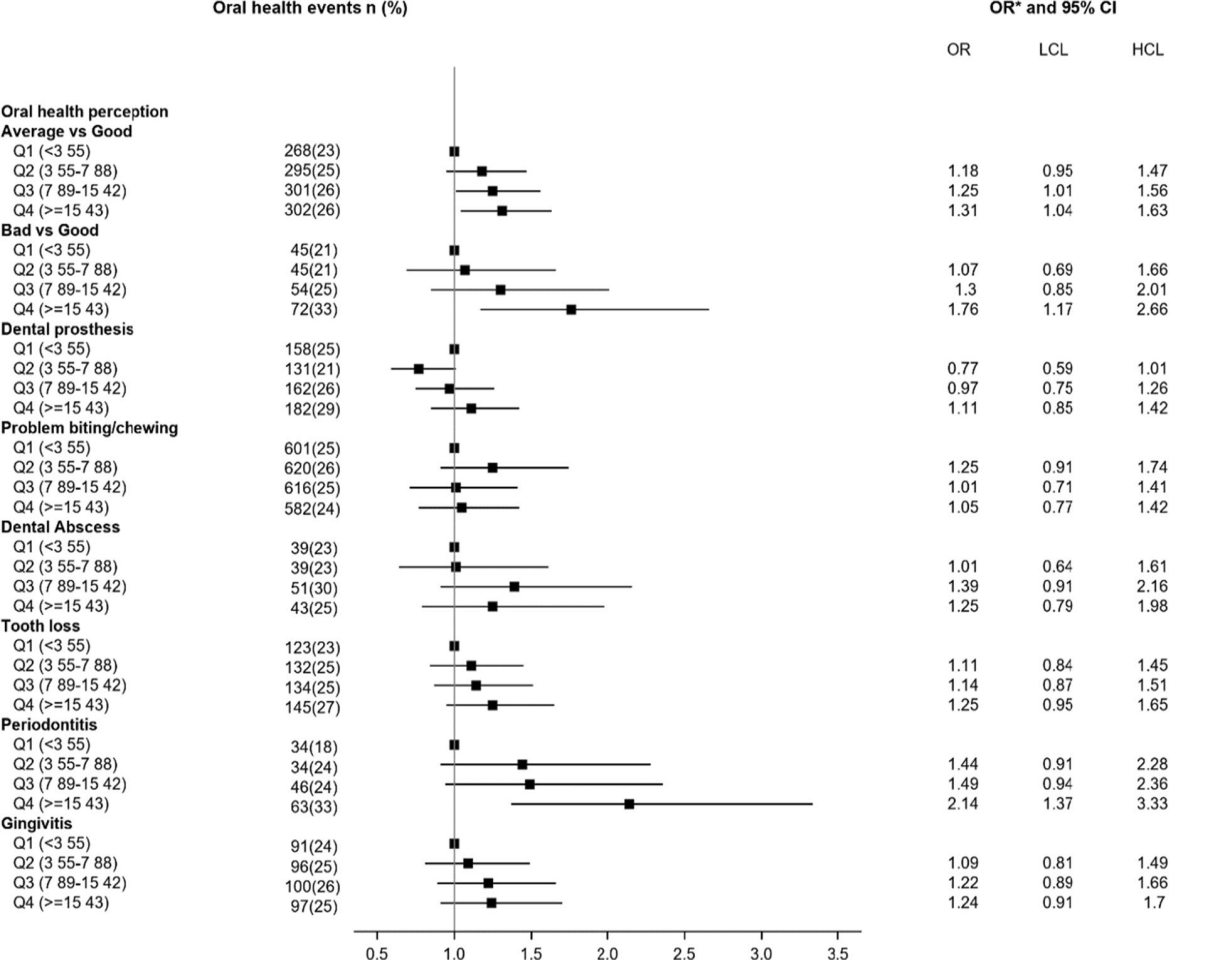
Odds ratios of perceived oral health according to type 2 diabetes duration. LCI, low confidence interval; UCI, upper confidence interval; Q, quartile of the diabetes duration, * Adjusted for age, educational level, smoking status, physical activity, family history of diabetes, hypercholesterolemia, hypertension, body mass index, dietary inflammatory index, daily brushing frequency and annual frequency of visit to the dentist

Figure 2 shows the adjusted OR of perceived oral health associated with the duration of type 2 diabetes. We observed a positive association between type 2 diabetes duration and poor perceived oral health. Women with a long duration of type 2 diabetes (in the 4th quartile group, ≥ 15 years) were more likely to report poor perceived oral health [adjusted OR = 1.76 (95% CI 1.17 to 2.66)] compared to women with short duration of type 2 diabetes (in the first quartile). With regards to components of oral health, women with type 2 diabetes for more than 15 years were more likely to report having periodontitis [adjusted OR = 2.14 (95% CI 1.37 to 3.33)], wearing dental prostheses [adjusted OR = 1.76 (95% CI 1.17 to 2.66)] and having lost teeth [adjusted OR = 1.25 (95% CI 0.95 to 1.65)] compared to women with short duration < 4 years (Figs. 2).

**Fig. 3:**
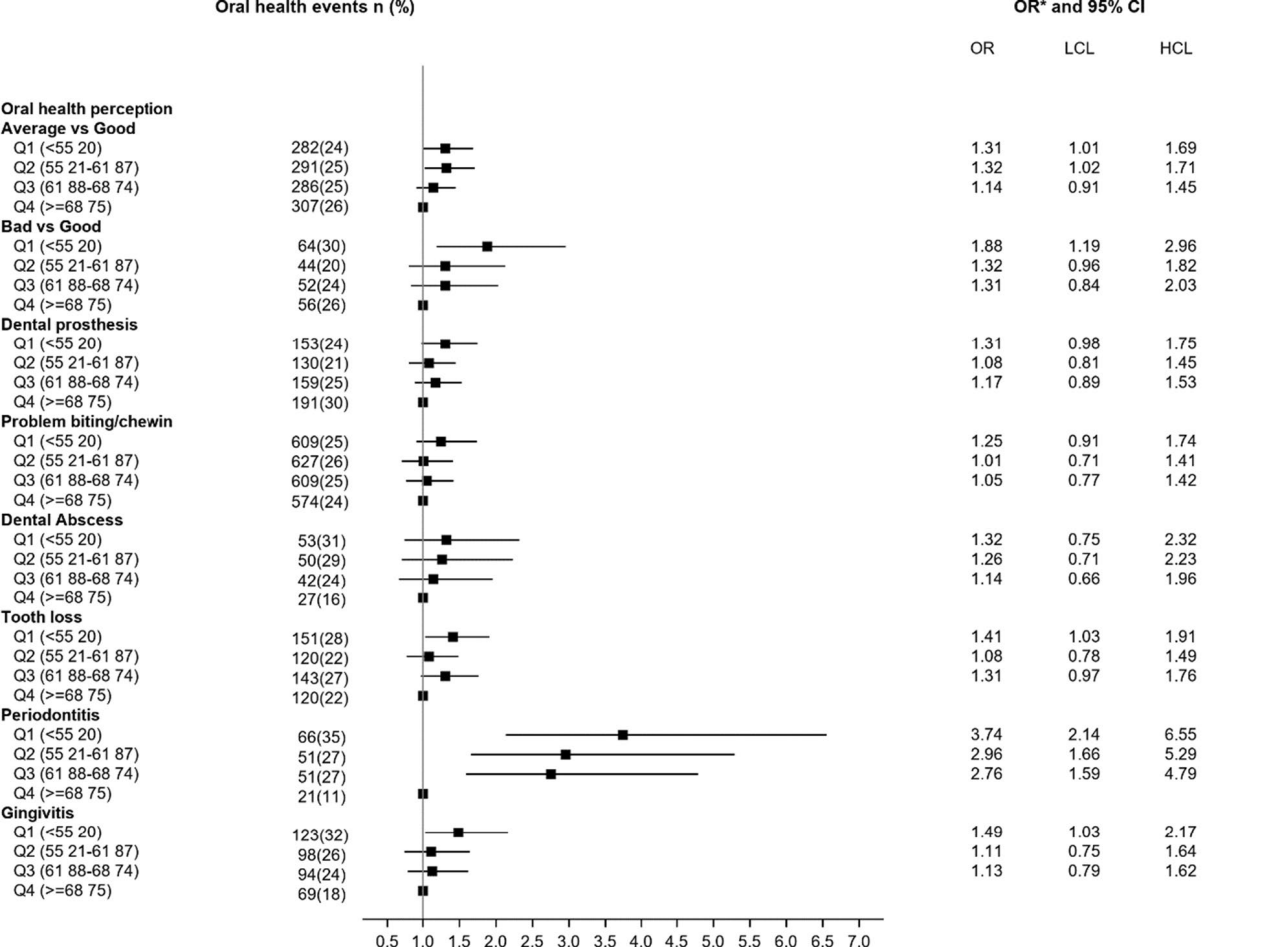
Odds ratios of perceived oral health according to age at diagnosis of type 2 diabetes. LCI, low confidence interval; UCI, upper confidence interval; Q, quartile of the diabetes duration, * Adjusted for age, educational level, smoking status, physical activity, family history of diabetes, hypercholesterolemia, hypertension, body mass index, dietary inflammatory index, daily brushing frequency and annual frequency of visit to the dentist

The same patterns of association were observed when age at diagnosis of type 2 diabetes was considered. Compared to women diagnosed at age 69 year or above (in the fourth quartile group), those diagnosed before the age of 55 year were more likely to report poor perceived oral health (Fig. 3). In addition, their odds of reporting gingivitis were higher [adjusted OR = 1.49 (95% CI 1.03 to 2.17)].

### Type 2 diabetes treatment and perceived oral health

Table 2 shows the adjusted OR of perceived oral health associated with types 2 diabetes treatment used. After adjustment for multiple potential confounding covariables, the likelihood of poor perceived oral health was higher for women with type 2 diabetes with no pharmacological treatment [OR = 1.34 (95% CI 1.08 to 1.67)] or for those with current use of OAD [OR = 1.24 (95% CI 0.99 to 1.57)] or insulin [OR = 2.33 (95% CI 1.27 to 4.29)] or both OAD and insulin [OR = 2.47 (95% CI 1.38 to 4.42)] compared to women without type 2 diabetes (Table 2). With regards to components of oral health, women using OAD and insulin were more likely to report wearing dental prostheses [adjusted OR = 2.05 (95% CI 1.36 to 3.09)] and having loose teeth [adjusted OR = 1.80 (95% CI 1.18 to 2.75)] compared to women without type 2 diabetes. In the fully adjusted model, the types of treatment were not associated with reporting dental abscesses, periodontitis and gingivitis.

**Table 2:** Adjusted Odds ratios of self-reported oral health associated with types 2 diabetes treatment use (N = 60,590 women), the E3N study

Oral health characteristics	Number (%)		Model^a^		Model^b^	
			OR	95% CI	OR	95% CI
**Oral health perception**	**Good**	**Average**				
Women without type 2 diabetes	32,932 (95.71)	21,879 (94.94)	1.00	Reference	1.00	Reference
Women with type 2 diabete**s**						
No pharmacological treatment	702 (2.04)	541 (2.35)	**1.16**	**1.04–1.30**	1.06	0.95–1.19
Oral antidiabetic (OAD)	665 (1.93)	525 (2.28)	**1.19**	**1.06–1.33**	1.07	0.95–1.20
Insulin	57 (0.17)	59 (0.26)	**1.56**	**1.08–2.24**	**1.47**	**1.02–2.12**
OAD and insulin	56 (0.16)	42 (0.18)	1.13	0.76–1.69	1.00	0.66–1.48
	**Good**	**Poor**				
Women without type 2 diabetes	32,932 (95.71)	2922 (93.12)	1.00	Reference	1.00	Reference
Women with type 2 diabetes						
No pharmacological treatment	702 (2.04)	100 (3.19)	**1.61**	**1.30–1.99**	**1.34**	**1.08–1.67**
OAD	665 (1.93)	89 (2.84)	**1.51**	**1.21–1.89**	**1.24**	**0.99–1.57**
Insulin	57 (0.17)	13 (0.41)	**2.57**	**1.41–4.70**	**2.33**	**1.27–4.29**
OAD and insulin	56 (0.16)	15 (0.48)	**3.03**	**1.71–5.36**	**2.47**	**1.38–4.42**
**Dental prosthesis**	**No**	**Yes**				
Women without type 2 diabetes	49,107 (95.67)	8626 (93)	1.00	Reference	1.00	Reference
Women with type 2 diabetes						
No pharmacological treatment	1048 (2.04)	295 (3.20)	**1.60**	**1.41–1.83**	**1.26**	**1.10–1.45**
OAD	1000 (1.95)	279 (3.00)	**1.59**	**1.39–1.82**	**1.21**	**1.05–1.40**
Insulin	105 (0.20)	24 (0.30)	1.30	0.84–2.03	1.11	0.70–1.75
OAD and insulin	76 (0.15)	37 (0.40)	**2.77**	**1.87–4.11**	**2.05**	**1.36–3.09**
**Problems of biting and chewing**	**No**	**Yes**				
Women without type 2 diabetes	51,011 (95.47)	6722 (93.88)	1.00	Reference	1.00	Reference
Women with type 2 diabetes						
No pharmacological treatment	1132 (2.12)	211 (2.95)	**1.42**	**1.22–1.64**	**1.24**	**1.06–1.44**
OAD	1089 (2.04)	190 (2.65)	**1.32**	**1.13–1.55**	1.14	0.97–1.34
Insulin	110 (0.21)	19 (0.27)	1.31	0.81–2.14	1.21	0.74–1.99
OAD and insulin	95 (0.18)	18 (0.25)	1.44	0.87–2.38	1.23	0.74–2.06
**Dental abscess**	**No**	**Yes**				
Women without type 2 diabetes	54,208 (95.28)	3525 (95.35)	1.00	Reference	1.00	Reference
Women with type 2 diabetes						
No pharmacological treatment	1263 (2.22)	80 (2.16)	0.97	0.78–1.22	0.94	0.75–1.19
OAD	1208 (2.12)	71 (1.92)	0.90	0.71–1.15	0.89	0.69–1.14
Insulin	116 (0.20)	13 (0.35)	1.72	0.97–3.06	1.72	0.96–3.06
OAD and insulin	105 (0.18)	8 (0.22)	1.17	0.57–2.41	1.40	0.55–2.36
**Tooth loss**	**No**	**Yes**				
Women without type 2 diabetes	46,950 (95.29)	10,783 (95.28)	1.00	Reference	1.00	Reference
Women with type 2 diabetes						
No pharmacological treatment	1081 (2.19)	262 (2.32)	1.06	0.92–1.21	1.11	0.96–1.27
OAD	1063 (2.16)	216 (1.91)	0.89	0.76–1.03	0.97	0.83–1.13
Insulin	102 (0.21)	27 (0.24)	1.15	0.75–1.76	1.14	0.74–1.75
OAD and insulin	83 (0.17)	30 (0.27)	**1.58**	**1.04–2.39**	**1.80**	**1.18–2.75**
**Periodontitis**	**No**	**Yes**				
Women without type 2 diabetes	53,040 (95.21)	4693 (96.13)	1.00	Reference	1.00	Reference
Women with type 2 diabetes						
No pharmacological treatment	1260 (2.26)	83 (1.70)	**0.75**	**0.60–0.93**	0.84	0.67–1.06
OAD	1189 (2.13)	90 (1.84)	0.86	0.69–1.06	1.05	0.84–1.31
Insulin	120 (0.22)	9 (0.18)	0.85	0.43–1.67	0.89	0.45–1.76
OAD and insulin	105 (0.19)	8 (0.16)	0.86	0.42–1.77	1.13	0.55–2.34
**Gingivitis**	**No**	**Yes**				
Women without type 2 diabetes	49,660 (95.26)	8073 (95.46)	1.00	Reference	1.00	Reference
Women with type 2 diabetes						
No pharmacological treatment	1150 (2.21)	193 (2.28)	1.03	0.89–1.21	1.10	0.94–1.29
OAD	1124 (2.16)	155 (1.83)	0.85	0.72–1.01	0.94	0.79–1.12
Insulin	106 (0.20)	23 (0.27)	1.34	0.85–2.10	1.42	0.90–2.23
OAD and insulin	99 (0.19)	14 (0.17)	0.87	0.50–1.52	0.99	0.56–1.75

Data in bold indicate statistically significant associations

CI, Confidence interval; OR, Odds ratios; OAD, Oral antidiabetic treatment

^a^Model 1: unadjusted

^b^Model 2: adjusted for age at wave 10, educational level, smoking status, physical activity, family history of diabetes, hypercholesterolemia, hypertension, body mass index, dietary inflammatory index, daily brushing frequency and annual frequency of visits to the dentist

There was no interaction between diabetes status and diabetes characteristics and BMI, smoking status and family history of diabetes on oral health. Further adjustment for mentally tiring work did not change the results (data not tabulated).

## Discussion

The present study demonstrates that older women with diabetes were more likely to report poor oral health than those without diabetes. Diabetes characteristics such as age at diabetes diagnosis, diabetes duration and diabetes treatment were also correlated with poor perceived oral health, even after adjusting for multiple potential confounders. With regards to components of oral health, diabetes duration and age at diagnosis were correlated with periodontitis and gingivitis in addition to wearing prostheses and problems of biting and chewing.

In the general population, oral health has been associated with low socioeconomic status and social security coverage [27]. Although in our study, all women were affiliated to the French national health insurance plan for teachers and our analyses took into account the level of education, we found an association between diabetes status and oral health. The high risk we observed would probably be higher in French women of the same age in the general population.

It has been found that individuals with diabetes have a higher prevalence or higher risk of oral health in several cross-sectional and prospective studies [28–30]. The main oral diseases explored so far in the literature were periodontal disease and caries. For periodontal disease, the odds is increased approximately threefold in people with diabetes compared to the general population [31] and for dental caries, people with type 2 diabetes exhibit higher rates of dental caries and are at higher risk of caries developing [32, 33].

There are very few epidemiological studies directed to tooth loss, wearing prostheses and problems of biting and chewing, and studies are exclusively focused on the associations with diabetes status [4] and glycemic control [29, 30, 34–36]. Kapp et al. reported a correlation between diabetes and tooth loss in a population-based sample of adults [36]. A recent study in French population also reported that people with diabetes tend to undergo dental extractions earlier and more often compared to those without diabetes [29].

Few studies have considered the different characteristics of diabetes such as duration, age at diagnosis in relation to oral health, and none have included as many parameters as those included in this study. Three papers have previously shown an association between the duration of diabetes and a higher prevalence of oral health diseases [12, 37, 38]. Mohamed et al. reported that the level of coronal caries was significantly higher in long compared to short duration of type 2 diabetes group (≤ 10 years) [12]. In addition, Moore et al. reported that longer duration of diabetes was possibly related to partial tooth loss [37] and to periodontal disease [38]. Similarly, we have not been able to find any studies in the literature regarding the treatments used for diabetes in relation with oral health.

As first described by Seifert in 1862 and in recent studies, diabetes is a metabolic disorder with several manifestations that are also perceivable in the oral cavity [39, 40]. These manifestations include aberrant evolution of dentition, increased prevalence of caries and pathologies of the oral mucosa [32].

Considering the mechanisms that explain the high prevalence of poor overall oral health perception and tooth loss in women with diabetes compared to those without diabetes is the high glucose level in gingival crevicular fluid and saliva among diabetic patients, which favors the diffusion of microorganisms on the tooth surface [41, 42]. This accumulation of microorganisms accelerates the microbial accumulation by lowering the executing tendency of neutrophils, and then maximizes the odds of developing tooth decay among people with diabetes [32]. In addition, it has been suggested that there is a degree of synergism between diabetes and oral diseases such as periodontal diseases [28, 43]. Inflammation plays a central role in both periodontal diseases and diabetes. Inflammatory processes are controlled in the periodontal tissues of people with diabetes and, on the other hand, the presence of periodontal diseases can have an effect on the metabolic control in diabetes, explaining thus, in part, the bidirectional relationship of these two diseases [43]. In the fully adjusted model, we did not find any association between type 2 diabetes status and inflammatory dental diseases (dental abscesses, periodontitis and gingivitis). Interestingly, long duration and early age at diagnosis of type 2 diabetes were related to these inflammatory dental diseases. This may suggest that more severe diabetes or comorbidities were involved in the association between diabetes and periodontal diseases. In addition, diabetes duration and age at diagnosis are likely associated with metabolic control. It may explain the research showing a greater rate of people with poor glycemic control in people who have periodontitis compared with people with diabetes who do not have periodontitis, as well as the research showing improvement in glycemic control after periodontal therapy in people with diabetes [44].

Our study presents several strengths. First, the E3N study included a large number of participants, and the large number of participants with type 2 diabetes ensured a high statistical power, however some analyses where the number of participants with specific oral disease was more limited and should be interpreted with caution. Second, we simultaneously evaluated in the same population, various oral health components in relation with diabetes status and diabetes characteristics. Finally, we were able to adjust our analyses for numerous risk factors as potential confounders, including daily brushing of teeth and annual frequency of visits to the dentist. Our study also has limitations. First, the study is cross-sectional and cannot provide information regarding causality nor temporality. Second, the lack of data on HbA1c level and ethnicity/race prevents us from adjusting our analyses for these variables. Third, the use of non-validated self-reported questionnaires to assess oral health could lead to a risk of bias, we believe that there is a limited risk of having a differential bias between the two groups compared, namely women with and without diabetes.. Moreover, in our study, 98% of the women reported visiting a dentist regularly; which increases the reliability of the diagnosis of the events like periodontitis and gingivitis which can be diagnosed only by a professional. Fourth, not all covariables were obtained from the 10th wave questionnaire. However, availability of data at E3N cohort study recruitment and over the follow-up allowed us to incorporate measurement of the closest wave. Fifth, we could not include all the population because of the missing data on one or more oral health items, however, the 60,590 women included did not differ from the 9152 others in terms of age, sociodemographic characteristics, and risk factors. Seventhly, our study population was exclusively composed of women; however, this limitation should have been minor because no difference in biological mechanisms has been reported between men and women regarding the influence of diabetes and its characteristics on oral health. Finally, our results are not directly generalizable to the French population because the E3N cohort is composed of women who were affiliated to the French national health insurance plan for teachers, the *Mutuelle Générale de l'Education Nationale*. They therefore represent a selected population of French women. However, it has been previously shown that an important socio-demographic gradient exists in the E3N cohort study which ensures a high diversity in the profiles of the study participants [45].

## Conclusion

The results of our study contribute to fill the gap in the existing literature by showing an association between type 2 diabetes and oral diseases, independent of daily brushing of teeth and the frequency of visits to the dentist. We have shown that diabetes duration and age at diagnosis are cross-sectionally associated with wearing prostheses, problems of biting and chewing, periodontitis and gingivitis. Type 2 diabetes treatments which provide some idea of the severity of the diabetes are also associated with oral health characteristics except inflammatory dental diseases. Given the predicted increase in the prevalence of diabetes, our results suggest a need for specific prevention strategies to preserve oral health in this at-risk population.

## Supplementary Information

**Additional file 1. Table s1:** Characteristics of the E3N study population according to diabetes duration (N = 60,590). **Table s2:** Characteristics of the E3N study population according to age at diabetes diagnosis (N = 60,590). **Table s3:** Characteristics of the E3N study population according to diabetes treatment (N = 60,590). **Figure s1**: Flowchart representing the constitution of the study sample.

## Data Availability

The datasets generated during and/or analysed for the current study are available from the corresponding author on reasonable request.

## Abbreviations

CNIL: Commission for Computerized Data and Individuals
E3N: Etude Epidémiologique auprès de femmes de l'Education Nationale
T2D: Type 2 diabetes
CI: Confidence interval
OR: Odds ratios
OAD: Oral antidiabetic treatment
ESM: Electronic supplementary material
WHO: World Health Organization
SD: Standard deviation
SAS: Statistical analysis systems
