# Supplementary material for: Type 2 diabetes and its characteristics are associated with poor oral health: findings from 60,590 senior women from the E3N study
Source: BMC Oral Health. 2021 Jun 23;21:315. doi: 10.1186/s12903-021-01679-w (PMC8220760; doi:10.1186/s12903-021-01679-w)
Supplement: Supplementary file 1 — Additional file 1. Table s1: Characteristics of the E3N study population according to diabetes duration (N = 60,590). Table s2: Characteristics of the E3N study population according to age at diabetes diagnosis (N = 60,590). Table s3: Characteristics of the E3N study population according to diabetes treatment (N = 60,590). Figure s1: Flowchart representing the constitution of the study sample. [file 12903_2021_1679_MOESM1_ESM.docx]

**Type 2 diabetes and its characteristics are associated with poor oral health: findings from 60,590 senior women from the E3N study**

Nasser LAOUALI,^1,2^; Douae EL FATOUHI,^1,2^; Gloria AGUAYO,^3^; Beverley BALKAU,^4^; Marie-Christine BOUTRON-RUAULT,^1,2^; Fabrice BONNET,^5^; Guy FAGHERAZZI^1,2,3^

**Running Title: Type 2 diabetes and oral health**

**Affiliations**

1: Center for Research in Epidemiology and Population Health (CESP), Inserm (Institut National de la Santé et de la Recherche Médicale) U1018, Generations and Health, Gustave Roussy Institute, 114 rue Edouard Vaillant, 94805 Villejuif Cedex, France

2: Faculté de Médecine, UPS-UVSQ-Paris-Saclay University, 94270 Le Kremlin-Bicêtre Cedex, France

3: Deep Digital Phenotyping Research Unit, Department of Population Health, Luxembourg Institute of Health (LIH), Strassen, Luxembourg

4: Center for Research in Epidemiology and Population Health (CESP), Inserm (Institut National de la Santé et de la Recherche Médicale) U1018, Clinical Epidemiology, 16 Avenue Paul Vaillant Couturier, 94807 Villejuif, France

5: CHU Rennes, Université de Rennes 1, Department of Endocrinology, Diabetology and Nutrition, Rennes, France

Supplementary Figure

**ESM Fig. 1**

E3N participants who had completed the 10th wave questionnaire

(n = 70,592)

Missing data on one or more oral health items

(n = 9,152)

All Type 2 Diabetes cases that occurred after the 10th wave questionnaire return (n = 852)

Final study population

(n = 60,590)

**ESM Fig. 1:** Flowchart representing the constitution of the study sample

Supplementary tables

Table s1: Characteristics of the E3N study population according to diabetes duration (N=60,590)

|  | **Women without type 2 diabetes (N=57,733)** | **Quartile (Q) of type 2 diabetes duration (years)** | | | |
| --- | --- | --- | --- | --- | --- |
|  |  | **Q1 (<4)**  **(N=714)** | **Q2 (4-8)**  **(N=714)** | **Q3 (8-15)**  **(N=715)** | **Q4 (≥15)**  **(N=714)** |
| Age (years) | 70.07 (6.20) | 69.71 (5.75) | 69.21 (5.77) | 71.86 (5.07) | 78.30 (4.10) |
| Educational level (%) |  |  |  |  |  |
| Undergraduate or less | 6,315 (10.94) | 129 (18.07) | 125 (17.51) | 112 (15.66) | 110 (15.41) |
| Graduate | 30,497 (52.82) | 385 (53.92) | 389 (54.48) | 373 (52.17) | 395 (55.32) |
| Postgraduate or more | 20,921 (36.24) | 200 (28.01) | 200 (28.01) | 230 (32.17) | 209 (29.27) |
| Physical activity (MET h/week) | 59.88 (47.86) | 50.75 (43.18) | 54.55 (44.23) | 55.93 (43.58) | 51.26 (44.81) |
| Smoking status (%) |  |  |  |  |  |
| Never | 2,748 (4.76) | 29 (4.06) | 26 (3.64) | 34 (4.76) | 18 (2.52) |
| Former | 15,788 (27.35) | 228 (31.93) | 199 (27.87) | 191 (26.71) | 164 (22.97) |
| Current | 39,197 (67.89) | 457 (64.01) | 489 (68.49) | 490 (68.53) | 532 (74.51) |
| Dietary inflammatory index | 0.14 (3.26) | -0.77 (3.20) | -0.29 (3.54) | -0.05 (3.00) | -0.54 (3.39) |
| BMI (kg/m^2^) | 23.86 (3.78) | 27.84 (6.08) | 28.00 (5.41) | 27.70 (5.38) | 26.57 (4.63) |
| Hypercholesterolemia (%) | 6,372 (11.04) | 94 (13.17) | 115 (16.11) | 139 (19.44) | 110 (15.41) |
| Hypertension (%) | 9,056 (15.69) | 219 (30.67) | 205 (28.71) | 213 (29.79) | 235 (32.91) |
| Family history of diabetes (%) | 6,672 (11.56) | 267 (37.39) | 230 (32.21) | 161 (22.52) | 125 (17.51) |
| Daily brushing (%) |  |  |  |  |  |
| Less than once | 631 (1.09) | 19 (2.66) | 24 (3.36) | 27 (3.78) | 23 (3.22) |
| One | 11,505 (19.93) | 212 (29.69) | 198 (27.73) | 194 (27.13) | 189 (26.47) |
| Two | 28,190 (48.83) | 339 (47.48) | 368 (51.54) | 331 (46.29) | 305 (42.72) |
| Three or more | 17,407 (30.15) | 144 (20.17) | 124 (17.37) | 163 (22.80) | 197 (27.59) |
| Visits to the dentist (%) |  |  |  |  |  |
| Once a year or more | 41,358 (71.64) | 511 (71.57) | 498 (69.75) | 479 (66.99) | 454 (63.59) |
| Sometimes | 15,234 (26.38) | 188 (26.33) | 195 (27.31) | 210 (29.37) | 223 (31.23) |
| Never visit to the dentist (%) | 1,141 (1.98) | 15 (2.10) | 21 (2.94) | 26 (3.64) | 37 (5.18) |

Table s2: Characteristics of the E3N study population according to age at diabetes diagnosis (N=60,590)

|  | **Women without type 2 diabetes (N=57,733)** | **Quartile (Q) of age at diabetes diagnosis (years)** | | | |
| --- | --- | --- | --- | --- | --- |
|  |  | **Q1 (<55)**  **(N=714)** | **Q2 (55-62)**  **(N=715)** | **Q3 (62-69) (N=714)** | **Q4 (≥69)**  **(N=714)** |
| Age (years) | 70.07 (6.20) | 71.66 (6.26) | 71.61 (6.37) | 72.19 (6.39) | 73.63 (6.18) |
| Educational level (%) |  |  |  |  |  |
| Undergraduate or less | 6,315 (10.94) | 101 (14.15) | 115 (16.08) | 128 (17.93) | 132 (18.49) |
| Graduate | 30,497 (52.82) | 388 (54.34) | 369 (51.61) | 403 (56.44) | 382 (53.50) |
| Postgraduate or more | 20,921 (36.24) | 225 (31.51) | 231 (32.31) | 183 (25.63) | 200 (28.01) |
| Physical activity (MET h/week) | 59.88 (47.86) | 55.59 (44.54) | 53.89 (42.18) | 55.42 (48.17) | 47.59 (40.27) |
| Smoking status (%) |  |  |  |  |  |
| Never | 2,748 (4.76) | 32 (4.48) | 26 (3.64) | 26 (3.64) | 23 (3.22) |
| Former | 15,788 (27.35) | 182 (25.49) | 206 (28.81) | 200 (28.01) | 194 (27.17) |
| Current | 39,197 (67.89) | 500 (70.03) | 483 (67.55) | 488 (68.35) | 497 (69.61) |
| Dietary inflammatory index | 0.14 (3.26) | -0.32 (3.36) | -0.33 (3.25) | -0.16 (3.40) | -0.85 (3.13) |
| BMI (kg/m^2^) | 23.86 (3.78) | 27.24 (5.03) | 27.84 (5.41) | 27.92 (5.71) | 27.10 (5.49) |
| Hypercholesterolemia (%) | 6,372 (11.04) | 122 (17.09) | 107 (14.97) | 121 (16.95) | 108 (15.13) |
| Hypertension (%) | 9,056 (15.69) | 228 (31.93) | 189 (26.43) | 225 (31.51) | 230 (32.21) |
| Family history of diabetes (%) | 6,672 (11.56) | 150 (21.01) | 156 (21.82) | 230 (32.21) | 247 (34.59) |
| Daily brushing (%) |  |  |  |  |  |
| Less than once | 631 (1.09) | 23 (3.22) | 22 (3.08) | 26 (3.64) | 22 (3.08) |
| One | 11,505 (19.93) | 190 (26.61) | 183 (25.59) | 211 (29.55) | 209 (29.27) |
| Two | 28,190 (48.83) | 318 (44.54) | 348 (48.67) | 328 (45.94) | 349 (48.88) |
| Three or more | 17,407 (30.15) | 183 (25.63) | 162 (22.66) | 149 (20.87) | 134 (18.77) |
| Visits to the dentist (%) |  |  |  |  |  |
| Once a year or more | 41,358 (71.64) | 495 (69.33) | 479 (66.99) | 474 (66.39) | 494 (69.19) |
| Sometimes | 15,234 (26.38) | 199 (27.87) | 208 (29.09) | 212 (29.69) | 197 (27.59) |
| Never visit to the dentist (%) | 1,141 (1.98) | 20 (2.80) | 28 (3.92) | 28 (3.92) | 23 (3.22) |

Table s3: Characteristics of the E3N study population according to diabetes treatment (N=60,590)

|  |  | **Women with type 2 diabetes (N=2,857)** | | | |
| --- | --- | --- | --- | --- | --- |
|  | **Women without type 2 diabetes (N=57,733)** | **No pharmacological treatment (N=1346)** | **Oral antidiabetic (N=1279)** | **Insulin (N=129)** | **OAD and insulin (N=113)** |
| Age (years) | 70.07 (6.20) | 71.91 (6.42)) | 72.60 (6.31) | 72.10 (6.31)) | 72.83 (5.76) |
| Educational level (%) |  |  |  |  |  |
| Undergraduate or less | 6,315 (10.94) | 243 (18.09) | 191 (14.93) | 20 (15.50) | 23 (20.35) |
| Graduate | 30,497 (52.82) | 676 (50.34) | 735 (57.47) | 72 (55.82) | 64 (56.64) |
| Postgraduate or more | 20,921 (36.24) | 424 (31.57) | 353 (27.60) | 37 (28.68) | 26 (23.01) |
| Physical activity (MET h/week) | 59.88 (47.86) | 55.00 (45.55) | 51.13 (40.32)) | 62.17 (61.65) | 42.92 (36.67) |
| Smoking status (%) |  |  |  |  |  |
| Never | 2,748 (4.76) | 63 (4.69) | 32 (2.50) | 9 (6.98) | 3 (2.65) |
| Former | 15,788 (27.35) | 369 (27.48) | 352 (27.52) | 29 (22.48) | 32 (28.32) |
| Current | 39,197 (67.89) | 911 (67.83) | 895 (69.98) | 91 (70.54) | 78 (69.03) |
| Dietary inflammatory index | 0.14 (3.26) | -0.30 (3.29) | -0.44 (3.37) | -0.94 (2.99)) | -0.87 (2.87) |
| BMI (kg/m^2^) | 23.86 (3.78) | 27.09 (5.15) | 27.92 (5.43) | 25.65 (5.97) | 30.29 (6.34) |
| Hypercholesterolemia (%) | 6,372 (11.04) | 227 (16.90) | 206 (16.11) | 13 (10.08) | 15 (13.27) |
| Hypertension (%) | 9,056 (15.69) | 413 (30.75) | 383 (29.95) | 39 (30.23) | 39 (34.51) |
| Family history of diabetes (%) | 6,672 (11.56) | 324 (24.13) | 396 (30.96) | 32 (24.81) | 32 (28.32) |
| Daily brushing (%) |  |  |  |  |  |
| Less than once | 631 (1.09) | 40 (2.98) | 43 (3.36) | 2 (1.55) | 8 (7.08) |
| One | 11,505 (19.93) | 346 (25.76) | 387 (30.26) | 28 (21.71) | 34 (30.09) |
| Two | 28,190 (48.83) | 622 (46.32) | 608 (47.54) | 64 (49.61) | 53 (46.90) |
| Three or more | 17,407 (30.15) | 335 (24.94) | 241 (18.84) | 35 (27.13) | 18 (15.93) |
| Visits to the dentist (%) |  |  |  |  |  |
| Once a year or more | 41,355 (71.64) | 910 (67.75) | 858 (67.08) | 99 (76.74) | 78 (69.03) |
| Sometimes | 15,230 (26.38) | 387 (28.82) | 371 (29.01) | 29 (22.48) | 33 (29.20) |
| Never visit to the dentist (%) | 1,141 (1.98) | 46 (3.43) | 50 (3.91) | 1 (0.78) | 2 (1.77) |
